# Supplementary material for: Causal Relationship between Aging and Anorexia Nervosa: A White-Matter-Microstructure-Mediated Mendelian Randomization Analysis
Source: Biomedicines. 2024 Aug 16;12(8):1874. doi: 10.3390/biomedicines12081874 (PMC11351342; doi:10.3390/biomedicines12081874)
Supplement: Supplementary file 1 [file biomedicines-12-01874-s001.zip › Supplementary Figure/Figure S4/Singe SNP plot of MR analysis for TL to ubm-b-1701.pdf]

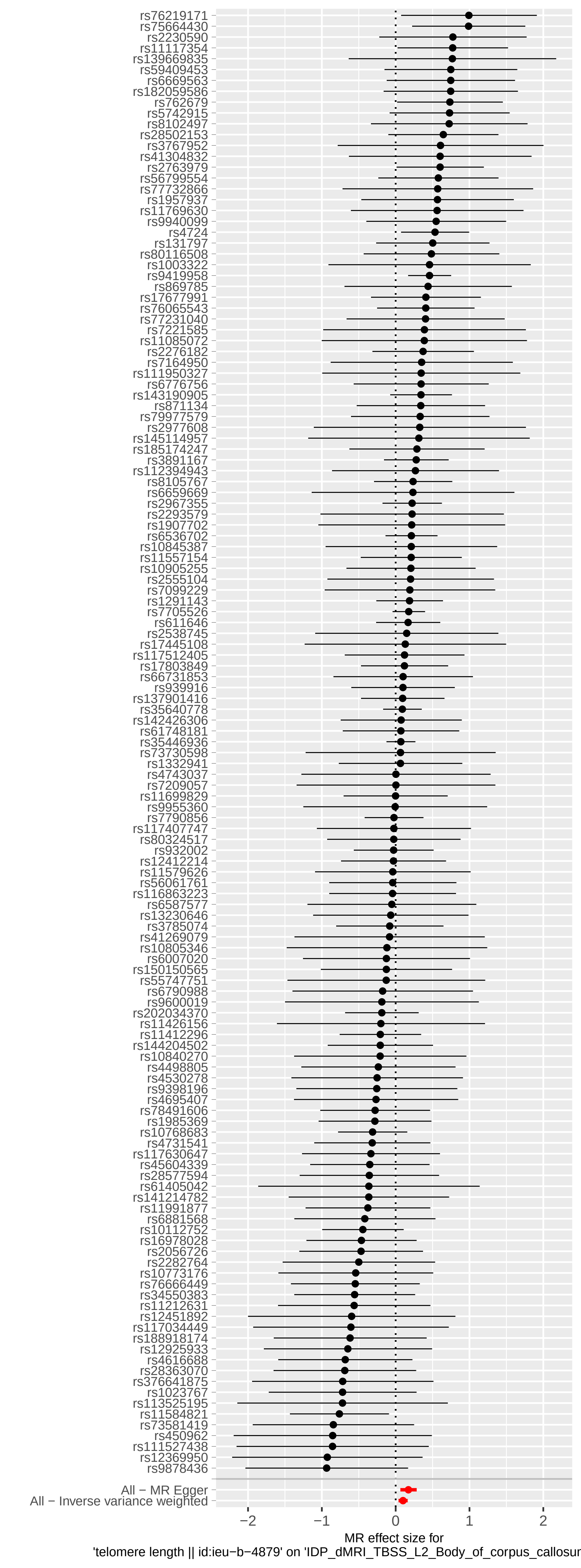
rs76219171
rs75664430
rs2230590
rs11117354
rs139669835
rs59409453
rs6669563
rs182059586
rs762679
rs5742915
rs8102497
rs28502153
rs3767952
rs41304832
rs2763979
rs56799554
rs77732866
rs1957937
rs11769630
rs9940099
rs4724
rs131797
rs80116508
rs1003322
rs9419958
rs869785
rs17677991
rs76065543
rs77231040
rs7221585
rs11085072
rs2276182
rs7164950
rs111950327
rs6776756
rs143190905
rs871134
rs79977579
rs2977608
rs145114957
rs185174247
rs3891167
rs112394943
rs8105767
rs6659669
rs2967355
rs2293579
rs1907702
rs6536702
rs10845387
rs11557154
rs10905255
rs2555104
rs7099229
rs1291143
rs7705526
rs611646
rs2538745
rs17445108
rs117512405
rs17803849
rs66731853
rs939916
rs137901416
rs35640778
rs142426306
rs61748181
rs35446936
rs73730598
rs1332941
rs4743037
rs7209057
rs11699829
rs9955360
rs7790856
rs117407747
rs80324517
rs932002
rs12412214
rs11579626
rs56061761
rs116863223
rs6587577
rs13230646
rs3785074
rs41269079
rs10805346
rs6007020
rs150150565
rs55747751
rs6790988
rs9600019
rs202034370
rs11426156
rs11412296
rs144204502
rs10840270
rs4498805
rs4530278
rs9398196
rs4695407
rs78491606
rs1985369
rs10768683
rs4731541
rs117630647
rs45604339
rs28577594
rs61405042
rs141214782
rs11991877
rs6881568
rs10112752
rs16978028
rs2056726
rs2282764
rs10773176
rs76666449
rs34550383
rs11212631
rs12451892
rs117034449
rs188918174
rs12925933
rs4616688
rs28363070
rs376641875
rs1023767
rs113525195
rs11584821
rs73581419
rs450962
rs111527438
rs12369950
rs9878436
All − MR Egger
All − Inverse variance weighted
−2
−1
0
1
2
MR effect size for
'telomere length || id:ieu-b-4879' on 'IDP_dMRI_TBSS_L2_Body_of_corpus_callosur
